# Outcomes of patients with stage I–II Hodgkin lymphoma who had uniform pre-treatment staging with PET/CT and treatment with limited field radiation therapy after chemotherapy

**DOI:** 10.1038/s41408-022-00711-8

**Published:** 2022-08-22

**Authors:** Kelsey M. Frechette, Scott C. Lester, Kekoa Taparra, William G. Breen, James A. Martenson, Bradford S. Hoppe, Jennifer L. Peterson, William G. Rule, Scott L. Stafford, Bradley J. Stish, Thomas M. Habermann, Jason R. Young, William S. Harmsen, Nadia N. Laack

**Affiliations:** 1 Department of Radiation Oncology, Mayo Clinic, Rochester, MN USA; 2 Department of Radiation Oncology, Stanford Medicine, Palo Alto, CA USA; 3 Department of Radiation Oncology, Mayo Clinic, Jacksonville, FL USA; 4 Department of Radiation Oncology, Mayo Clinic, Scottsdale, AZ USA; 5 Department of Hematology, Mayo Clinic, Rochester, MN USA; 6 Department of Radiology, Mayo Clinic, Rochester, MN USA; 7 Division of Biomedical Statistics and Informatics, Mayo Clinic, Rochester, MN USA

**Keywords:** Radiotherapy, Radiotherapy

## Introduction

Combined modality therapy (CMT) consisting of chemotherapy followed by radiation therapy (RT) is an accepted standard of care for stage I-II Hodgkin lymphoma (HL). The German Hodgkin Study Group trials HD7-HD11 [1] helped to establish the parameters for CMT, ultimately leading to HD10 and HD11 and adoption of 20 Gy and 30 Gy, using involved field RT (IFRT), as the standard dose of RT following ABVD for favorable and unfavorable HL, respectively. Relapse has not to be eliminated, however. For example, the HD10 trial reported a 10 year progression-free survival rate of 87% [1].

The target volume in IFRT includes lymph nodes involved by HL and uninvolved nodes in the same lymph node group. Results from the British Columbia Cancer Agency provided evidence that the size of RT fields used in CMT could be reduced to involved-node RT (INRT), targeting only nodes involved at initial staging [2]. INRT reduces the dose of radiation to normal organs [3]. A study in non-Hodgkin lymphoma provided evidence that INRT reduces late toxicity, compared to IFRT [4].

INRT fields are appropriate with optimal pre-chemotherapy imaging [5], allowing fusion of a staging PET/CT scan to a post-chemotherapy RT planning CT scan. When a PET/CT in RT planning position is not done, larger involved site RT (ISRT) fields are used to allow for greater variation in the position of involved nodes [5].

PET/CT has been shown to upstage 14% of patients compared with CT staging [6]. Pretreatment PET/CT was shown to modify RT planning in 17.7% of patients in one study [7]. The identification of otherwise unappreciated areas of involvement by pre-treatment PET/CT may reduce the risk of relapse. This retrospective study assessed relapse and other outcomes in stage I-II HL, staged with pre-treatment PET/CT followed by CMT that included consolidative RT using IFRT or ISRT.

## Methods and materials

This study had Institutional Review Board approval. Patients with biopsy-proven stage I–II HL treated at the Mayo Clinic in Minnesota with CMT from 2000 to 2011 were identified. Eligibility criteria included a pre-treatment PET/CT performed at our institution, and a complete response (CR) or partial response (PR) to first line chemotherapy, followed by IFRT or ISRT. Patients with unfavorable HL [8], including those with bulky disease, were eligible. A mediastinal mass ≥1/3 the trans-thoracic diameter or any mass ≥10 cm was considered bulky [9, 10]. All patients were imaged to assess post-chemotherapy response with a PET/CT or CT scan. Assessment of response was based on the Lugano Criteria [10].

RT fields were retrospectively classified as ISRT or IFRT, as described by the International Lymphoma Radiation Oncology Group [5] and Yahalom and Mauch [11], respectively. Pre-treatment PET/CT imaging was not done in RT planning position. Accordingly, no patient was treated with INRT.

Symptomatic toxicity attributable to RT was retrospectively assessed using Common Terminology for Adverse Events, version 5.0. Assessment of thyroid toxicity also included asymptomatic abnormally increased thyroid stimulating hormone (TSH). Adverse events occurring during RT, or within one month of completion of RT, were defined as acute toxicity. All other adverse events were defined as post-radiation toxicity.

January 31, 2018 was the cutoff for all endpoints. Survival and relapse were measured from start of treatment to death from any cause and relapse, respectively. Survival was estimated using the Kaplan-Meier method. The incidence of relapse, with death as a competing risk, was calculated using the cumulative incidence function. The 95% confidence interval (95% CI) was calculated at 5 and 10 years for survival and relapse. All statistical analyses used SAS version 9.4 and R version 3.6.2.

## Results

Ninety-six patients were eligible. Consent for medical record-based research was declined by 3. The remaining 93 patients form the basis of this study.

Patient characteristics are summarized in the Table. Unfavorable disease was present in 52 patients (56%) [9]. The Median follow-up was 7.5 years.

ABVD was used in 86 patients (92%) (Table 1). Thirty patients (32%) received 1–3 cycles of chemotherapy and the remainder received 4–6 cycles. The dose of radiation was 20–21 Gy in 55 patients (59%), >21 to ≤30 Gy in 32 (34%) and >30 Gy in 6 (6%). Three dimensional conformal RT was used in 66 patients (71%) and intensity modulated RT was used in 27 (29%). ISRT was used in 84 patients (90%), and IFRT in 9 (10%).

**Table 1:** Patient characteristics.

Patient characteristics	Number of patients (%) *n* = 93
**Ann Arbor Stage at diagnosis**	
IA	14 (15%)
IB	1 (1%)
IIA	65 (70%)
IIB	13 (14%)
**Favorable/Unfavorable risk**	
Favorable	40 (43%)
Unfavorable	53 (57%)
**Bulky Disease**	
≥ 1/3 TTD	49 (53%)
≥ 10 cm mass	29 (31%)
**Histology**	
Nodular sclerosis	75 (81%)
Mixed cellularity	3 (3%)
Lymphocyte-rich	1 (1%)
Lymphocyte-depleted	0 (0%)
Classical Hodgkin lymphoma, not otherwise specified	8 (9%)
Nodular lymphocyte predominant	6 (6%)
**Sex**	
Female	48 (52%)
Male	45 (48%)
**Age at diagnosis**	
0–20 years old	14 (15%)
21–40 years old	52 (56%)
41–60 years old	20 (22%)
≥61 years old	7 (8%)

*TTD* trans-thoracic diameter.

Eighty-nine patients were evaluated for response following chemotherapy by PET/CT: 84 (94%) had a CR and 5 (6%) had a PR. All 4 patients evaluated by CT following chemotherapy had a CR.

Survival and cumulative incidence of recurrence are shown in the Figure.

**Fig. 1:**
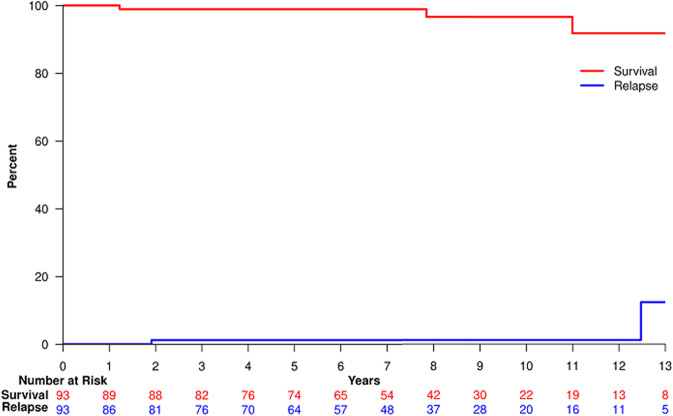
Survival and cumulative incidence of recurrence. Overall survival (red) and cumulative incidence of recurrence (blue).

Overall survival at 5 and 10 was 98.9% (95% CI 96.5–100%) and 96.6% (95% CI 90.3–100%).Three patients died, all of causes that did not appear to be related to prior RT (Fig. 1). Heart failure was the cause of death in a 74-year-old who had a history of this problem before RT. Cor pulmonale was the cause of death in a 71 year old whose RT fields were limited to the right neck. An 88-year-old patient treated with RT to the left axilla and supraclavicular area died of aortic stenosis and dementia.

The cumulative incidence of recurrence at 5 and 10 years was 1.2% (95% CI 0.2–8.5%). Two relapses occurred, both with disease in prior RT fields.

The most common acute toxicity was esophagitis, documented in 45 patients (48%), with 44 and 1 patients experiencing grade 2 and 3 symptoms, respectively. Eleven patients (12%) experienced grade 1 (10 patients) or grade 2 (1 patient) acute skin toxicity. One patient experienced acute grade 2 oral mucositis and one experienced grade 3 oral mucositis.

The most common post-RT toxicity was hypothyroidism. No post-RT thyroid toxicity was observed in the 13 patients who did not have thyroid gland in the radiation fields. In the remaining 80 patients hypothyroidism was documented in 28 (35%) with 8 and 20 patients experiencing grade 1 and 2 toxicity, respectively. There was one case each of multi-nodular goiter, benign thyroid nodule and Hashimoto’s thyroiditis.

All other acute and post-RT toxicities occurred with a frequency of less than 3%, and in no case did any other toxicity exceed grade 2.

Six patients were diagnosed with post-treatment second malignancies, excluding non-melanoma skin cancers: Ewing sarcoma at 1.7 years, follicular lymphoma at 3.2 years, prostate cancer at 5.4 years, melanoma at 5.8 years, chronic lymphocytic leukemia at 11.1 years, and multiple myeloma at 13.8 years.

## Discussion

The rate of recurrence was very low in patients with stage I–II HL who were uniformly staged with pre-treatment PET/CT and then treated with CMT. These results were achieved despite a high burden of unfavorable disease, including 52% with bulky mediastinal involvement and 31% with masses ≥10 cm. The low rate of recurrence is consistent with randomized [8] and non-randomized studies [12, 13] that suggest that consolidative RT mitigates the adverse effect of bulky disease. These favorable also suggest that the potential benefit of reduced toxicity with smaller RT fields need not come with an increased risk of relapse.

Thyroid toxicity occurred exclusively in patients with thyroid gland in the radiation fields. Consistent with the landmark study from Stanford [14], post-RT thyroid toxicity was common in our study: 35% of patients with thyroid gland in the radiation fields experienced hypothyroidism. Stanford reported a higher rate of thyroid toxicity, reflecting longer follow-up in their study. The incidence of hypothyroidism would likely be higher in the present study with additional follow-up. These findings reinforce the importance of evaluation of TSH during follow-up in RT patients treated with thyroid gland in RT fields.

Most prospective studies have appropriately confined assessment of RT-related toxicity to patients with grade 3 or higher adverse events. In the 20 and 30 Gy arms of the HD10 trial, for example, the rate of grade 3–4 gastrointestinal toxicity (including esophageal toxicity) was 2.9% and 5.7% respectively [15]. In our study 48% experienced grade 2 (44 patients) or grade 3 (1 patient) acute esophagitis. Our study complements prospective studies by providing a more comprehensive assessment of the burden of toxicity attributable to RT.

The retrospective nature of this study and the resulting heterogeneity in treatment parameters are important limitations. Most patients treated with ABVD, for example, received more than 3 cycles. In the absence of a prospective protocol, and with evolving treatment standards, it was not possible to determine consistent reasons for treatment heterogeneity. The possibility that the favorable results in our study were at least in part attributable to more intensive treatment with ABVD cannot be excluded.

## Conclusions

A low rate of relapse was observed in this study of patients with stage I–II HL who were uniformly staged with PET/CT and then treated with CMT. Excellent outcomes were achieved despite a high burden of bulky disease (52% of patients) and the use of limited radiation fields. These results provide support for pre-treatment staging with PET/CT followed by CMT using limited RT fields. Hypothyroidism was observed in 35% of patients with the thyroid gland in the radiation fields, reinforcing the importance of assessment of TSH during follow-up in this group.

## Data Availability

Data will be shared with researchers who provide a methodologically sound proposal, subject to Institutional Review Board approval, and subject to any limitations placed by the Institutional Review Board.
